# PeerOnCall: Evaluating Implementation of App-Based Peer Support in Canadian Public Safety Organizations

**DOI:** 10.3390/ijerph22081269

**Published:** 2025-08-13

**Authors:** Sandra E. Moll, Rosemary Ricciardelli, R. Nicholas Carleton, Joy C. MacDermid, Stephen Czarnuch, Renée S. MacPhee

**Affiliations:** 1 School of Rehabilitation Science, McMaster University, Hamilton, ON L8S 4L8, Canada; 2 School of Maritime Studies, Fisheries and Marine Institute, Memorial University, St. John’s, NL A1C 5R3, Canada; rricciardell@mun.ca; 3 Department of Psychology, University of Regina, Regina, SK S4S 0A2, Canada; nick.carleton@uregina.ca; 4 School of Physical Therapy, University of Western Ontario, London, ON N6A 3K7, Canada; jmacderm@uwo.ca; 5 Department of Electrical and Computer Engineering, Memorial University, St. John’s, NL A1C 5S7, Canada; sczarnuch@mun.ca; 6 Department of Kinesiology & Physical Education, Wilfrid Laurier University, Waterloo, ON N2L 3C5, Canada; rmacphee@wlu.ca; 7 Department of Health Sciences, Wilfrid Laurier University, Waterloo, ON N2L 3C5, Canada

**Keywords:** m-health, first responder, mental health, implementation science

## Abstract

Public safety personnel (PSP), including correctional workers, firefighters, paramedics, police, and public safety communicators, are at increased risk for posttraumatic stress injury, yet face barriers in receiving timely support. Mobile health (mHealth) applications (apps) offer promising avenues for confidential, on-demand access to relevant information and support. The purpose of this study was to assess implementation of *PeerOnCall*, a new mHealth platform designed by and for PSP (the platform includes two parallel apps: one for frontline workers and one for peer support providers). A multi-site mixed methods implementation trial was conducted over 3−6 months in 42 public safety organizations across Canada. App usage trends were tracked through software analytics, and facilitators and barriers to app use were explored via interviews with organizational champions. Over 11,300 employees across 42 organizations were invited to use the *PeerOnCall* app over the trial period, with approximately 1759 PSP (15% of total) downloading the app. Variation within and across sectors was evident in app downloads and feature use. Approaches to communication (mode, timing, and messenger), and organizational culture related to mental health and help outreach affected uptake levels. *PeerOnCall* is a promising tool to facilitate access to peer support; however, culturally relevant strategies are needed to overcome barriers and integrate this tool into workplace practices.

## 1. Introduction

Public safety personnel (PSP; [1]) include border services, correctional workers, firefighters, paramedics, police officers, and public safety communicators (communicators, e.g., 911 call-takers, dispatchers). PSP are essential service providers who play a foundational role in keeping communities safe [2]. The work of PSP inherently involves repeated exposures to potentially psychologically traumatic events (PPTEs; [1]) that increase their risk for posttraumatic stress injuries (PTSIs) relative to the general public [3]. There is also significant evidence that other operational and organizational stressors negatively impact PSP mental health [4,5]. Internationally, qualitative work with PSP in the United Kingdom who were exposed to trauma at work noted the “pervasive, cumulative, and salient impact of occupational trauma on mental health”, including negative impacts from job demands, insufficient and inadequate support, and stigma, all of which interfered with outreach for mental health support [6].

Despite increased PTSI risks, PSP often refrain from seeking or receiving timely help [7,8]. Barriers to support include the stigma associated with seeking help, fear of professional repercussions, and lack of access to service providers who understand their work context [5,9]. Access barriers are greater for PSP working in rural or remote areas or PSP who have limited access to workplace benefits (e.g., volunteer firefighters). Insufficient access to evidence-based resources exacerbates mental health risks for the individual, their families, their colleagues, and the communities they serve [6].

Digital technologies such as mobile health (mHealth) applications (apps) offer promising avenues for accessing interventions and support [10]. Mobile apps provide opportunities for on-demand (24/7), convenient access to relevant information and support. Widespread use of smartphones makes mHealth apps scalable, cost-effective tools for communicating mental health information and support, and may reduce the stigma associated with seeking mental health support [11,12]. There are now more than 20,000 mental health apps that are currently on the market [11]. The relatively small number of available mobile health apps tailored for PSP continues to grow. A scoping review identified 22 apps designed specifically to build mental health resilience among the military, veterans, and PSP [13]. Most of the identified apps included components of mindfulness training, psychoeducation, cognitive behavioral therapy, and/or acceptance and commitment therapy; however, the quality varied widely for tools, content, and associated research evidence [13].

Research regarding the impact of digital mental health interventions (i.e., apps, wearables, and web-based programs) on diverse users is growing, including studies specifically examining their effectiveness in workplace contexts. Several workplace mental health review studies have reported small beneficial effects of digital mental health interventions for reducing symptoms of depressive-, anxiety-, and trauma-related disorders, and general stress [14,15,16]; reducing insomnia and burnout [14,15]; and improving psychological well-being [17]. A recent meta-analysis supported a small but statistically significant sustained effect from digital mHealth interventions on employee engagement and productivity outcomes [16].

The promising elements of digital mental health interventions for workplace mental health are offset by concerns about low engagement, high dropout rates, and challenges with sustaining outcomes over time [18,19,20]. A review of apps for the PSP community indicated that half of the apps had not been tested in randomized trials, only 68% used evidence-based strategies or components, and dose–response rates were unaddressed [13]. There are growing calls to examine how the workplace environment influences the adoption of technology, with contextual factors either supporting or hindering employee uptake, use, and overall impact [21,22]. Exclusive focus on symptom reduction without analyzing implementation processes may preclude an understanding of the relative levels of impact based on the app, the app content, or workplace context [20].

The current study was designed to examine the implementation of *PeerOnCall*, a new mobile health platform (a pair of interconnected apps with one used by PSP peer supporters, and one used by PSP seeking support) co-created with PSP and designed to promote early intervention and access to peer support. It is different from existing workplace apps due to its primary focus on early intervention and peer support, and its focus on the unique experience of public safety providers [23]. The study objectives are to (1) identify trends in app use among employees in participating organizations; (2) compare how the app was implemented within and across the participating organizations; and (3) identify facilitators and barriers to app use.

## 2. Materials and Methods

### 2.1. Design

A concurrent explanatory mixed-methods implementation design (quant + QUAL) [24] was used to explore and explain *PeerOnCall* app use across multiple PSP organizations in correctional services, firefighting, paramedics, police, and public safety communicators. The multi-organizational case study implementation design provided an opportunity to gather quantitative data on app use trends in the real-world context of PSP organizations alongside qualitative data from organizational champions to explain the factors potentially influencing these trends. We adopted an implementation science approach informed by the Consolidated Framework for Implementation Research (CFIR) to examine contextual forces shaping app use patterns [25]. The CFIR is a comprehensive and widely cited framework that provides a structured approach to understanding and analyzing implementation across five domains, including the outer setting, inner setting, innovation, people, and implementation process [26].

The current study synthesized data from two parallel *PeerOnCall* implementation studies conducted between May 2022 and March 2024. The core interdisciplinary research team spanned faculty and graduate students from five Canadian universities, as well as knowledge users from across Canada. There were five PSP sectors included in the current research (i.e., communicators, corrections, fire, police, and paramedics), each supported by an academic lead and at least one knowledge user with a history of collaboration and program implementation expertise for their respective sector. The implementation period ranged from 3 to 4 months for 9 of the participating organizations and to 6 months for the remaining 33 organizations. The shorter time frame was due to implementation delays within several services that joined the trial at a later date.

This study was conducted in accordance with the Declaration of Helsinki, and the protocol was approved by the Hamilton Integrated Research Ethics Board (2022-14731-GRA) on 29 July 2022. Ethics approval was also obtained through review boards in three additional universities affiliated with the academic sector leads. Informed consent was obtained from all participants as per the project protocol. The project included an advisory team of public safety leaders, service providers, and knowledge users/stakeholders.

### 2.2. Recruitment

Organizations were recruited for app implementation using established PSP networks from among the research team members. Eligible organizations required (1) support from leadership to participate (e.g., signed organizational agreement letters); (2) at least one organization champion to liaise between the research team and employees; and (3) an internal peer support service with procedures for vetting, training, and ongoing support for the peer support providers. In several organizations that did not have an established peer support team, researchers supported the program implementation by providing training to establish or enhance the current peer support service within the organization. Organizations were purposively recruited to represent diversity in size and geographical location to facilitate comparison of implementation across a range of contexts. Differences in the number and size of organizations within each sector reflected differences in how services are organized and provided. For example, correctional services recruitment occurred provincially, fire service recruitment included volunteer, career, and composite (combined) services, and communications recruitment involved proactive outreach and combining organizations given resource limitations. Paramedic service recruitment included small, mid-sized, and large organizations in urban and rural/remote communities. Recruited police services included one large urban service and a smaller service in a more remote community.

Each organization identified at least one “champion” who volunteered to serve as a liaison between the organization and the research team. Champions included workplace wellness providers, managers, and peer support team leads. Their role was to facilitate communication with employees regarding the project, coordinate onboarding with the peer support providers, and to communicate with the research team regarding any concerns.

### 2.3. Intervention

*PeerOnCall* is an mHealth platform created to promote early intervention and support PSP well-being [2]. It was co-designed by and for the public safety community through a process of engagement with over 75 stakeholders across Canada and beta testing with 93 frontline PSP across three provinces [23]. The platform includes an app for frontline workers (i.e., *PeerOnCall*) that connects individuals to trained peer supporters within their organization, offering accessible, confidential, and secure peer support via text or phone, with protocols for crisis intervention. The app also provides access to over 80 original evidence-informed articles and peer-wisdom videos providing “tips to cope” from frontline PSP perspectives. Related features included links to local resources, instruments to track wellness and set goals, and the option to build a wellness toolbox of personal “grounding” images. Peer support providers used a companion app (i.e., *PeerOnCall Support*) with secure anonymous links for peer support provision and tools enabling peer supporters to indicate their availability, access resources to help support their work, and track encounters. The platform includes an administrative portal (*PeerOnCall Admin*) for organizational champions to upload local resources, customize content, send notifications to app users, update peer supporters, and track general app use over time.

The mHealth platform was initially set up within each organization for the internal peer support team to be visible to the app users. Accounts were created for the peer supporters on the *PeerOnCall Support* app. Peer supporters could then upload an image and short biography, and get oriented to the app technology, enabling them to respond to support requests. All initial connections were initiated via text by employees using the *PeerOnCall* app; they could choose from the list of available peer supporters within their organization by browsing the bios and sending an initial text message. Once a message is sent, the peer support provider receives an alert via the *PeerOnCall Support* app, and can respond via text (or enable a secure phone conversation). The peer support provider did not know the identity of the employee (only an ID number), and there were no limits on the number of phone or text messages that could be exchanged. The team also uploaded local resources relevant to the organization, thus customizing the app for each participating organization (e.g., the Employee Family Assistance Program [EFAP] contacts, wellness programming). All employees were then invited to download the *PeerOnCall* app and to participate in the research via established communication channels within the organization using a QR code to download the app and a unique organizational code to access the resources and services within the app. Organizational engagement approaches varied from initial to ongoing communications with employees about the mobile app and access to recruitment materials (e.g., posters, business cards, and promotional materials) including a QR code for research participation.

### 2.4. Data Collection

Data collection within each organization included software analytics to track app use patterns and baseline as well as one to two sets of follow-up interviews with organizational champions. All app use metrics were based on anonymous, aggregated data collected through the software platform. Interviews with organizational champions were designed to explore information about the workplace context, the implementation process, and perceived facilitators and barriers to app use. The interviews occurred in-person, by phone, or through a virtual platform, and were 20 to 90 min in length.

### 2.5. Data Analyses

Descriptive data about app use were collected over time, including basic demographic data of app users (age, gender, years of experience), number of downloads, number of app opens, access to app features, and outreach to peer support. Data were collected within each organization, with comparisons of trends across organizations, services, and sectors. Analysis of the interview and focus group transcripts was used to inform interpretation of the adoption and engagement data within and across participating organizations, services, and sectors. The CFIR framework informed an inductive analytic approach that considered broad categories related to the outer and inner context of the organizations, the people involved, responses to the technology, and the implementation process [24,27]. Coding was iterative, noting key facilitators and barriers to app engagement. The current paper focuses on factors that influenced the implementation process.

## 3. Results

### 3.1. Characteristics of App Users

We invited over 11,300 employees across 42 organizations to use the *PeerOnCall* app over the 3–6 month trial period. Organizations varied in size from 12 to 3000 employees. Most participating organizations (83%; *n* = 35) were small (i.e., under 200 employees). There were three medium-sized organizations (i.e., 200 to 750 employees) and four large-sized organizations (i.e., over 750 employees). Organizations were recruited from across Canada, including Atlantic Canada (*n* = 16), Ontario (*n* = 19), Quebec (*n* = 1), Western Canada (*n* = 5), and Nunavut (*n* = 1). Organizations were also recruited across the five public safety sectors including provincial correctional facilities across two provinces (*n* = 14); fire services (*n* = 14; including a mix of career, volunteer, and hybrid); paramedic services (*n* = 10); public safety communicators (*n* = 2); and police (*n* = 2).

**Figure 1:**
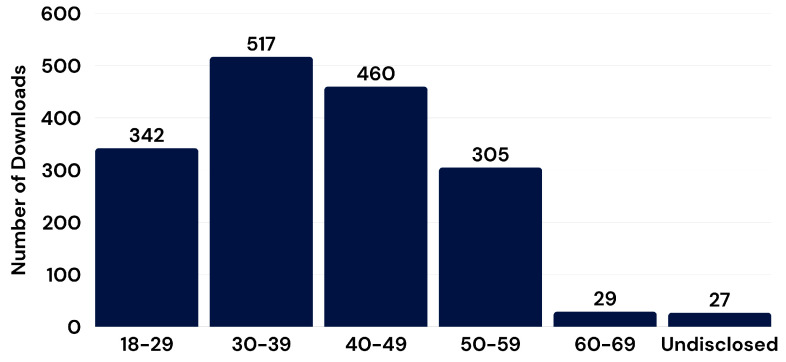
Number of app users by age (# of total downloads).

**Figure 2:**
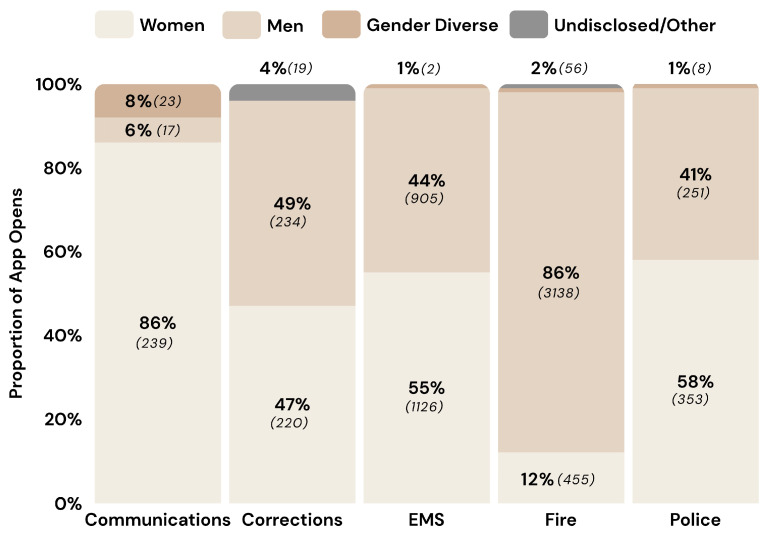
Percentage of app users across sectors by gender.

PSP who downloaded the app were asked to provide demographic data regarding their gender, age, and years of experience. Most downloads (i.e., 31%) were by users 30 to 39 years old (see Figure 1). There were gender differences across sectors (see Figure 2), such that public safety communicators had the highest proportion of women (i.e., 86%) and the fire sector had the lowest proportion of women (i.e., 12%).

### 3.2. App Use Trends

Approximately 1759 of the 11,401 PSP who were invited to participate (i.e., 15% of the total) downloaded the app on their phones during the study. Download proportions (see Table 1) were highest among public safety communicators (*n* = 54; 46% of invited participants) and lowest among police (*n* = 271; 8% of invited participants). There was considerable variability across organizations within each sector. For example, in the fire sector organizations, download proportions ranged from 12 to 100%, averaging 18%. In the paramedic sector, download proportions ranged from 9% to 74%, averaging 21%.

Most participants (i.e., 92%) who downloaded the app opened the app at least once. On average, each app user opened the app approximately four times over the course of the 3–6 month trial, with a range of one to 190 app opens per user. Approximately 31% of app users opened the app once, 60% opened it between two and ten times, and 9% opened it more than 10 times. Most app opens were during the first month after downloading (31%, *n* = 2188), with approximately 12% to 15% of downloads each month from months two to six. Implementation delays within some services due to securing permissions or completing training requirements meant that approximately 10 participating organizations only had a 3–4 month implementation period.

Most app opens were among participants who identified as men (i.e., 64%; *n* = 1047), with fewer app openings among participants who identified as women (i.e., 34%; *n* = 470) or as either “non-binary” or “prefer to self-describe” (i.e., 0.5%; *n* = 7). The gendered proportion of app users varied by sector (see Figure 1). More men than women used the app, but outreach to peer support was proportionally higher among women (i.e., 22%, *n* = 117 connections) than men (14%, *n* = 191 connections).

Individuals between the ages of 40 and 49 years had the highest proportion of app opens (i.e., 34%, *n* = 2421) and were the most likely to reach out for peer support (i.e., 20%). Participants who were 30–39 years old had the next highest proportion of app opens (i.e., 30%, *n* = 2100) and were the next most likely to reach out for peer support (i.e., 15%).

### 3.3. Qualitative Analyses

Interview data from organizational champions provided an opportunity to explore the considerable variability in app use by assessing the influence of the implementation process, as well as perceived facilitators and barriers to app engagement, as informed by the CFIR framework. Table 2 provides an overview of the number and timing of champion interviews in each sector (several organizations were represented by more than one champion). Of the 107 champion interviews, 57 were baseline (seven interviews were with more than one champion) and 50 were follow-up interviews (after 3–6 months).

Organizational champions provided many insights into the implementation process, as well as facilitators and barriers related to the outer context of PSP culture, the inner context of organizations (e.g., culture, resources, and work infrastructure), the perceived value of the app itself, and the influence of employee stakeholders. The findings presented here reflect the most significant facilitators and barriers to app use that were mentioned by champions across sectors and organizations (i.e., consistently noted across organizations and emphasized as important). Quotes from participants across sectors are provided to illustrate key points.

#### 3.3.1. Implementation Processes

Diverse communication modes were used across organizations to communicate with potential participants about the app. Each organization adopted its own strategy and established communication channels, including email notices, posters, in-person sessions, online orientations, and branded promotional materials (e.g., cards, mugs, water bottles, and pens). Communication materials provided the app website or associated QR code in order to download the app, plus a unique organizational code to open the app.

Most participants reported that posting communications on websites and sending email messages was the least effective way to increase app engagement. Champions across sectors explained that PSP rarely check their emails. One champion from the fire sector said the following: “*when you come with something new, a tool, but it’s not emphasized too much, it will pass like the rest, among the 15 others you have to read for the day.*” There was agreement among participants that in-person communication was the most effective. One paramedic said that “*nothing even comes close to face-to-face rollout for communication. I think it’s the only way that works.*” A public safety communicator suggested an email would be deleted, but “*if they’re sitting there viewing it and having someone explain it to them, then it’s maybe more well-received.*”

In-person connections were also recognized as very time-consuming and logistically difficult, particularly for multi-site organizations and for PSP who typically work across shifts. Many champions suggested that future communication strategies could include introducing the app during established orientation and training programs, particularly when onboarding new recruits. For example, one public safety communicator champion recommended “*if we are going to implement this in the future, this is something that I feel should be part of the training process.*” Champions across sectors emphasized the importance of having dedicated time to download and trial the technology.

Another consideration was the timing of the communication. In many organizations, the focus was on the initial launch, with limited ongoing communication. Organizations that included regular reminders and proactive follow-up led to higher rates of engagement.

Champions also noted that the source of the messaging was critical to encourage app uptake. For example, champions working in the police and fire sectors described the critical role of leaders in supporting app use, given the hierarchical nature of decision-making in their organization. Other sector champions described the importance of messaging from the “ground up” because peer supporters might be perceived as more trusted than management. One paramedic champion explained that “*it’s the trust and the faith they have in our peer support team. And I feel if they’re the ones that are pushing [PeerOnCall], a lot more individuals will be more supportive of that.*” Several champions described the value of a multi-pronged approach; for example, in one fire service organization, the app was launched with coordinated communication from leadership, the union, and the peer supporters. Another paramedic champion stated that “*it’s a combination of people that have different viewpoints on it; people they can trust. So not just one person but maybe a few different people with different angles.*”

Overall, the implementation process played a critical role in shaping uptake, considering the messenger as well as the medium and timing of the communication within each organization.

#### 3.3.2. Facilitators of App Use

Champions across sectors described key facilitators of app use, including the changing organizational culture (inner context) and the *PeerOnCall* app features.

Many organizational champions described the outer setting of PSP culture as changing with respect to mental health and the need to reach out for support. A fire participant reported, “*the culture is shifting which is good. It’s slowly shifting,*” explaining that there was a trend towards increased acceptance of mental health challenges and decreased stigma. Their observation was echoed by champions across sectors. A paramedic champion reported efforts to “*venture away from the ‘suck it up buttercup’ attitude*” and indicated “*that is changing and we’ve all been working quite hard at that.*” Overall, participants described cultural changes including increased receptivity to strategies and tools for supporting mental health and help-seeking.

All organizational champions highlighted specific features of the mobile health platform that were highly valued. Participants reported that the app was easy to use and helped to centralize information in one place. For example, a police champion explained the following: “*One thing the app has going for it is the ease of access. I mean, you don’t have to remember phone numbers or search through emails to find contact info. It’s all right there.*” Participants appreciated the information and resources provided by the app, including how the resources were easy to access and customized for PSP. Participants also appreciated how app users had a choice in when and how to access resources based on individual interests and needs. A public safety communicator champion noted how their colleagues were able to “*watch these videos which might resonate with them*”, explaining that this was helpful for those not ready to reach out for support.

Another highly valued app feature that was consistently identified was the opportunity for anonymous, confidential connections to support. A police participant explained that “*if they can communicate with a peer supporter by text completely anonymously, it takes some of that stigma away.*” A paramedic reported the app removes the “*awkwardness out of asking for help*”, explaining that “*you don’t have to look someone in the eye and admit you’re struggling. You can just type it out. The anonymity, the ability to reach out at any time, it’s revolutionary for us.*” Anonymity within the app was considered to be a critically important feature.

Participants across sectors hypothesized that younger PSP would prefer communicating through a cell phone with text-based options. For example, a police participant said that “*many of the new cops who are coming on were born with a cell phone in their hand, are tech-savvy, and prefer to communicate by text.*” A fire participant suggested “*…to give a tech option, like some of the young guys, So easy right?*” Using the app for peer support was considered an important option for help-seeking, particularly among younger PSP.

#### 3.3.3. Barriers to App Use

Participants described the outer setting of a changing PSP culture as a potential facilitator for app engagement, but there were many reports of resistance related to the long-standing culture of stoicism inherent to PSP work. A fire participant described the “superhero” mindset as preventing frontline workers from reaching out:

*“Some people won’t seek help, regardless of the resource or format. There’s also a culture of the strong man, the superhero who doesn’t need anyone and is supposed to provide help rather than seek it. They’re often accustomed to offering help rather than seeking it. So, this change in role is difficult.”*

Longer-serving PSP were noted to be more resistant to culture change, especially in the context of seeking support. A paramedic participant described younger PSP as “*more willing to actually ask for help and admit that they want to talk to somebody. So that’s a generational change that we’re seeing.*” There was also evidence of pervasive ongoing stigma in the inner setting associated with compromised mental health and fear of repercussions if others found out. Many participants reported that their peers raised questions about the anonymity associated with app use. There were concerns about being recognized by their voice or their story, even without providing their name. A corrections participant explained the following: “*The biggest impression that I’m getting from anybody that I talk to about it is they don’t believe it’s an anonymous app. … I think it’s just because we’re such a small organization. Like everybody knows everybody.*” A police participant echoed concerns about confidentiality and stigma and the potential for negative career implications if managers found out about their app use:

*“I think the biggest concern is convincing people this app isn’t something that’s being monitored by our department. There’s this underlying fear that maybe it’s tracking who’s using it or what’s being said. It’s not like a red flag is going to pop up on the inspector’s screen when someone talks to a peer supporter, but that’s what people worry about. They don’t want their struggles to be known by management, even though we’ve tried to assure them that the app is completely private. This fear has definitely kept some people from even downloading it.”*

In addition to concerns about privacy, practical barriers in the inner setting limited workplace access to the app. Participants working as public safety communicators, in corrections, and in some police services reported not being allowed to use a personal phone at work. A public safety communicator participant explained the following: “*I can’t put the app on my phone. So right away, big flag for me, so that means none of the communicators… because of the municipal or the city IT, or whatever security. So kind of disheartening because I know that my group or whatever won’t be able to use it.*” Some work policies precluded timely access to support through the app.

Participants across all sectors described delays and resistance to adoption of anything new. A paramedic participant reported how “*everything we’ve done in [EMS]… takes time. That’s one of the biggest things. There is no shortcut to getting people to change and getting people to use something. I’ve been doing this 14-years almost, and it’s been like every year there’s something and people complain as soon as there’s a change.*” A police participant similarly reported resistance to change, emphasizing skepticism and mistrust related to new technology:

*“There’s a real cultural hurdle we’re facing with this app. People are used to doing things a certain way, and introducing something new, especially something digital, is met with skepticism. …. But when there’s this underlying mistrust or lack of understanding, it’s hard to get people on board with any new technology, no matter how good it is.”*

Overall, the slow pace of culture change and policy restrictions in the outer and inner settings of PSP organizations were noted to be key barriers to the adoption of the *PeerOnCall* app.

## 4. Discussion

Research to date on engagement and use of app technology is limited [19], particularly in the PSP community. The current study provides important insights into *PeerOnCall* app implementation across a range of diverse organizations, considering how participants used the app as well as key facilitators and barriers to app use.

### 4.1. Patterns of App Use

The number of downloads and app opens varied widely across organizations, but the average download percentage overall was approximately 15%. The Technology Adoption Lifecycle (TAL) can provide a useful lens to contextualize the results. The TAL is an evidence-informed framework for understanding how people react to, adopt, and accept new innovative products and technologies [28]. Using the TAL curve, the 15% adoption proportion may represent the “innovator” (2.5%) and “early adopter” (13.5%) groups who are typically more open to trying technology innovations. Increasing usage beyond the group of early adopters is a “chasm” given the substantial challenges associated with engaging a broader “early majority” group [29]. The “early majority” group (approximately 34% of users) is typically pragmatic and data-driven, requiring proof of effectiveness as part of the engagement strategy [28,29]. Additional time and strategic efforts may be needed to engage a larger group of employees to achieve more mainstream adoption. Tailored strategies using diverse information sharing or app promotion strategies may be needed to match the perspective of a wider group of PSP and increase app uptake.

The pattern of initial engagement with the technology followed by a decrease over time is consistent with most review studies of user engagement in a “real world” context [10,19]. Patterns of user retention or the “stickiness” of the technology have been the subject of much speculation in the literature. Approximately 4% of users who download a mental health app continue using the app after 15 days, and 3% continue using the app after 30 days [30]. Diminishing user patterns are often described as “non-adoption” and/or “abandonment” of the technology, a problem that typically occurs over time [31]. There is a commonly held assumption that more frequent and continued use of an app over time is ideal; however, individual user needs and the purpose of an app influence adoption patterns [19,30]. Some users may have their needs addressed with fewer uses, whereas others may download an app anticipating future needs. For *PeerOnCall*, not every PSP requires support all the time, and we were unable to locate any research documenting typical rates of peer support outreach in person or online, precluding context for interpreting usage patterns.

Another important consideration is whether the number of downloads, usage, and connections should be the primary impact measures. For example, reviewing a peer-created information video about managing a bad call or reaching out for support might have positive short-term and/or long-term impacts that are difficult to measure. Measuring the impact of a single peer support connection is also difficult, in that the outreach might be for general reassurance, but might also prevent a suicide. Peer support may be less about the frequency of use and more about the qualitative impact on individual users. A prescribed number of app engagements is also inappropriate for assessing *PeerOnCall*, which is designed to be used as needed. Downloading and knowing how to use *PeerOnCall* means PSP can view the content, use the self-management tools, or reach out for peer support as per their own unique needs. Additional longitudinal research is needed to understand minimum use thresholds, as well as individual and organizational impacts over time.

App use trends in the current study suggest potentially important interactions with gender and age. More men than women downloaded the app, but a higher proportion of women reached out for peer support, which is consistent with research on gendered patterns of help-seeking [32]. The gender differences were small and may relate more to the gendered nature of PSP workplaces [33]. There were also trends related to age differences in download and peer outreach, with older participants (aged 30–49 years) being more likely to use the app, contrasting hypotheses made by organizational champions who predicted higher use among younger participants. Digital mental health intervention research provides mixed results with respect to the age of app users but generally indicates that engagement is highest among users 30 to 50 years old [18,19], noting that they may have higher expectations and more commitment to app use. In the current study focusing on the PSP community, involvement of leaders and established peer support providers in initial implementation may also contribute to higher levels of engagement, given that they reflect an older age group.

### 4.2. Facilitators and Barriers to App Use

Qualitative interview data helped to clarify factors impacting app use related to the implementation process. Differences in the nature of the messaging (e.g., content, format, and timing), messenger role and credibility, and resources dedicated to implementation may all explain some of the differences in uptake within and across the participating PSP sectors. Increasing evidence underscores the value and complexity of the implementation process on uptake of digital mental health interventions [18,21,31], with a growing number of tools for optimizing implementation [22,34]. The current results regarding the limited value of passive (e.g., email, posters) versus active engagement strategies (e.g., in-person onboarding sessions) are consistent with trends noted in the literature. For example, automated strategies (e.g., distributing educational materials and sending reminders) can help improve technology efficiency and expand reach but can appear impersonal and reduce levels of commitment [18,22]. Many studies emphasize that implementation success depends on a priori assessments of organizational readiness, leadership commitment, infrastructure, and resources [18].

Interview data also provided insights into perceived benefits and challenges related to *PeerOnCall*. Participants described *PeerOnCall* as easy to use with customizable content and attention to privacy, all of which are key features of quality app design emphasized in the extant literature [21,31]. Many app features were highly valued, but participants also indicated that the work environment was not necessarily conducive to supporting use of the app. Participants underscored the need to access their phone at work, as well as access to the time and space to privately reach out for support. PSP organizations need to carefully consider how existing policies and infrastructure might impact engagement and uptake to support successful implementation.

The study results also underscored the importance of establishing trust in introducing new technology within the PSP community. Participants reported that awareness, credibility, and trust all take time to build. Consistent with the extant literature [22,34], participants described endorsement from respected formal or informal leaders in the organization as critical for successful implementation. Participants continued to report stigma and reputational risks associated with help-seeking, which is congruent with results from a recent systematic review of barriers to mental health care among PSP [9]. The same review study underscored evidence of fears regarding confidentiality and negative career impact. The *PeerOnCall* app was designed to provide confidential, anonymous, and secure connections; nevertheless, there continued to be skepticism and fear that, at least in small communities, users could be identified. Addressing mental health stigma remains an important prerequisite for overcoming barriers to accessing e-mental health technology [22].

### 4.3. Recommendations for Implementation

Results of the analysis of the key issues shaping engagement with PeerOnCall within the public safety community point to the importance of tailored implementation strategies that take into account the unique context of PSP work. A summary of potential strategies to overcome each of the key barriers is outlined in Table 3.

### 4.4. Study Strengths, Limitations, and Future Directions

The current study has several strengths, including the breadth of implementation data gathered across multiple diverse PSP organizations. The results provide useful information about broad trends in implementation and uptake of the *PeerOnCall* app and meet a gap in the literature regarding app use in the public safety community. Limitations include the short time frame for implementation, particularly given the findings regarding the slow pace of change in public safety organizations. Longitudinal research (at least 6–12 months) is needed to track change over time. In addition, data were not collected from app users. Future research is needed to explore the perspective of frontline app users (and non-users), and how app use affects outcomes such as mental health, perceived support, and work performance. Another limitation is that the focus was on observing trends rather than studying specific approaches to implementation. Future research is needed to systematically evaluate implementation strategies designed to increase adoption (e.g., moving beyond the early adopters to “early majority” users), with sufficient time to track changes. An implementation protocol is also needed to incorporate lessons learned from this initial implementation study as well as contemporary research evidence into occupational e-mental health interventions.

## 5. Conclusions

Overall, the current study provides important insights into the implementation of *PeerOnCall*, a mobile health peer support platform co-designed by and for PSP. By identifying patterns of app use, organizational implementation, and facilitators and barriers to adoption, our results add to the growing body of evidence informing app implementation in real-world contexts. Advancing e-mental health technologies for PSP requires a nuanced understanding of the unique organizational context of their work and tailored evidence-based implementation strategies to achieve optimal outcomes.

## Figures and Tables

**Table 1:** Overview of app use by sector.

PSP Sector	Orgs (#)	Employees (#)	Downloads (#; %)	# of Times App Opened	Average # App Opens Per User
Public Safety Communications	2	118	54 (45.8)	279	5.16
Corrections	14	1117	131 (11.7)	473	3.61
Fire	14	4629	841 (18.2)	3649	4.34
Paramedic	10	2092	436 (20.8)	2039	4.67
Police	2	3320	271 (8.2)	612	2.26
Total	42	11,401	1759 (15.4)	7052	4.01

**Table 2:** Overview of champion interviews.

Sector	# Orgs	# Champions	# Baseline Interviews	# Follow-up Interviews
Communications	2	7	7	6 (3 mo) 4 (6 mo)
Corrections	14	12	12	7 (3 mo) 7 (6 mo)
EMS	10	24	14	3 (6 mo)
Fire	14	21	21	12 (3 mo) 11(6 mo)
Police	2	5	3	3
**Total**	42	55	57	50

**Table 3:** Strategies to optimize app engagement.

Barrier	Implementation Strategy
Communication channels	○ Integrate into existing initiatives (e.g., new recruit training, critical incident debriefings, wellness events) ○ Include hands-on practice and Q&A ○ Provide regular reminders (e.g., in-app notifications, updates in meetings) ○ Recruit “champions” from across the organization
Culture of stoicism	○ Educate on signs of burnout, moral injury, anxiety, and depression, and the importance of early intervention for faster recovery. Link to self-screening tools (accessible in app) ○ Reinforce that access to help in the app is anonymous and readily accessible ○ Share “use cases” of when and how the app could be used that incorporate local voices of trusted peers
Stigma and confidentiality concerns	○ Explain security and privacy features in the app ○ Share privacy policy and FAQs ○ Share protocol for protecting confidentiality in the context of peer support
Unable to use phone at work	○ Allocate dedicated time during orientation, huddles, or staff training sessions for downloading and navigating the app to ensure all staff are familiar with its features for use outside of work as needed ○ Revisit workplace policy to identify options for employees to comfortably access the app and/or peer support during work hours (e.g., mental health breaks); need time and private space ○ Explore how the app can be utilized within disability management processes and return-to-work strategies to assist workers who are isolated or struggling
Mistrust, resistance to change	○ Establish credibility through leadership endorsement ○ Leverage informal leaders or trusted peers as change champions to advocate and share positive experiences ○ Highlight quality, evidence-informed content that has been developed by and for PSP ○ Co-create new content that is customized to local needs and issues

## Data Availability

The datasets presented in this article are not readily available due to the complexity of the technical data regarding app use (required coding experience), and the sensitive nature of the qualitative data (organizations were assured of anonymity). Requests to access the datasets should be directed to the corresponding author (Sandra Moll; molls@mcmaster.ca).

## Abbreviations

The following abbreviations are used in this manuscript:

PSP	Public Safety Personnel
TAL	Technology Adoption Lifecyle
CFIR	Consolidated Framework for Implementation Research
mHealth	Mobile health
